# Supplementary material for: Prevalence of pulmonary tuberculosis among key and vulnerable populations in hotspot settings of Ethiopia. A systematic review and meta-analysis
Source: PLoS One. 2024 Aug 29;19(8):e0309445. doi: 10.1371/journal.pone.0309445 (PMC11361443; doi:10.1371/journal.pone.0309445)
Supplement: S1 File — (DOCX) [file pone.0309445.s001.DOCX]

**Prevalence of pulmonary tuberculosis among key and vulnerable populations in hotspot settings of Ethiopia. A Systematic Review and Meta-Analysis**

**Supplementary files**

**List of tables and figures**

[**S1 Table:** The databases, search strings, and recorded articles 1](#_Toc174820869)

[**S2 Table::** Quality appraisal results of included articles. 3](#_Toc174820870)

[**S3 Table:** The Egger’s publication bias test for small-study effects 4](#_Toc174820871)

[**S4 Table:** The nonparametric trim-and-fill analysis of publication bias 5](#_Toc174820872)

[**S1 Figure:** Funnel plot outcome for assessing publication bias. The prevalence of pulmonary TB is represented on the x-axis and the standard error (SE) of the prevalence of pulmonary TB is on the y-axis. 6](#_Toc174820707)

[**S2 Figure**: The Galbraith plots of outcome, showing the assessment of heterogenicity across studies. 6](#_Toc174820708)

**S1 Table:** The databases, search strings, and recorded articles

| **Database** | **Search terms** | **Result** |
| --- | --- | --- |
| **PubMed** | **Filters: Humans: Language: English; Date range: 01 January 2000 to 31 December 2023.**  ((((Prevalence) OR (epidemiology)) AND ((((((tuberculosis) OR (TB)) OR (pulmonary tuberculosis)) OR (*Mycobacterium tuberculosis*)) OR (*M. tuberculosis*)) OR (MTB))) AND (((((((((((((((congregate settings) OR (high-risk settings)) OR (prison)) OR (prisoner)) OR (prison inmate)) OR (jail)) OR (correctional facilities)) OR (Universities)) OR (Homeless shelters)) OR (Military camps)) OR (Refugee camps)) OR (Homeless shelters)) OR (Home for the elderly)) OR (Nursing homes)) OR (Healthcare settings))) AND (Ethiopia) Filters: Humans, English, from 2000/1/1 - 2023/12/31  (("epidemiology"[MeSH Subheading] OR "epidemiology"[All Fields] OR "prevalence"[All Fields] OR "prevalence"[MeSH Terms] OR "prevalance"[All Fields] OR "prevalences"[All Fields] OR "prevalence s"[All Fields] OR "prevalent"[All Fields] OR "prevalently"[All Fields] OR "prevalents"[All Fields] OR ("epidemiologies"[All Fields] OR "epidemiology"[MeSH Subheading] OR "epidemiology"[All Fields] OR "epidemiology"[MeSH Terms] OR "epidemiology s"[All Fields])) AND ("tuberculosi"[All Fields] OR "tuberculosis"[MeSH Terms] OR "tuberculosis"[All Fields] OR "tuberculoses"[All Fields] OR "tuberculosis s"[All Fields] OR "TB"[All Fields] OR ("tuberculosis, pulmonary"[MeSH Terms] OR ("tuberculosis"[All Fields] AND "pulmonary"[All Fields]) OR "pulmonary tuberculosis"[All Fields] OR ("pulmonary"[All Fields] AND "tuberculosis"[All Fields])) OR ("mycobacterium tuberculosis"[MeSH Terms] OR ("mycobacterium"[All Fields] AND "tuberculosis"[All Fields]) OR "mycobacterium tuberculosis"[All Fields]) OR ("*mycobacterium tuberculosis*"[MeSH Terms] OR ("mycobacterium"[All Fields] AND "tuberculosis"[All Fields]) OR "*mycobacterium tuberculosis"[*All Fields] OR "m tuberculosis"[All Fields]) OR "MTB"[All Fields]) AND ((("congregant"[All Fields] OR "congregants"[All Fields] OR "congregate"[All Fields] OR "congregated"[All Fields] OR "congregates"[All Fields] OR "congregating"[All Fields] OR "congregation"[All Fields] OR "congregation s"[All Fields] OR "congregational"[All Fields] OR "congregations"[All Fields]) AND ("setting"[All Fields] OR "setting s"[All Fields] OR "settings"[All Fields])) OR ("high-risk"[All Fields] AND ("setting"[All Fields] OR "setting s"[All Fields] OR "settings"[All Fields])) OR ("prison s"[All Fields] OR "prisoners"[MeSH Terms] OR "prisoners"[All Fields] OR "prisoner"[All Fields] OR "prisons"[MeSH Terms] OR "prisons"[All Fields] OR "prison"[All Fields]) OR ("prison s"[All Fields] OR "prisoners"[MeSH Terms] OR "prisoners"[All Fields] OR "prisoner"[All Fields] OR "prisons"[MeSH Terms] OR "prisons"[All Fields] OR "prison"[All Fields]) OR ("prisoners"[MeSH Terms] OR "prisoners"[All Fields] OR ("prison"[All Fields] AND "inmate"[All Fields]) OR "prison inmate"[All Fields]) OR ("jails"[MeSH Terms] OR "jails"[All Fields] OR "jail"[All Fields]) OR ("correctional facilities"[MeSH Terms] OR ("correctional"[All Fields] AND "facilities"[All Fields]) OR "correctional facilities"[All Fields]) OR ("universiti"[All Fields] OR "universities"[MeSH Terms] OR "universities"[All Fields] OR "university"[All Fields] OR "university s"[All Fields]) OR ("ill housed persons"[MeSH Terms] OR ("ill housed"[All Fields] AND "persons"[All Fields]) OR "ill housed persons"[All Fields] OR ("homeless"[All Fields] AND "shelters"[All Fields]) OR "homeless shelters"[All Fields]) OR (("militaries"[All Fields] OR "military personnel"[MeSH Terms] OR ("military"[All Fields] AND "personnel"[All Fields]) OR "military personnel"[All Fields] OR "military"[All Fields] OR "military s"[All Fields]) AND ("camp s"[All Fields] OR "camped"[All Fields] OR "camping"[MeSH Terms] OR "camping"[All Fields] OR "camps"[All Fields])) OR ("refugee camps"[MeSH Terms] OR ("refugee"[All Fields] AND "camps"[All Fields]) OR "refugee camps"[All Fields]) OR ("ill housed persons"[MeSH Terms] OR ("ill housed"[All Fields] AND "persons"[All Fields]) OR "ill housed persons"[All Fields] OR ("homeless"[All Fields] AND "shelters"[All Fields]) OR "homeless shelters"[All Fields]) OR (("home environment"[MeSH Terms] OR ("home"[All Fields] AND "environment"[All Fields]) OR "home environment"[All Fields] OR "home"[All Fields]) AND ("aged"[MeSH Terms] OR "aged"[All Fields] OR "elderly"[All Fields] OR "elderlies"[All Fields] OR "elderly s"[All Fields] OR "elderlys"[All Fields])) OR ("nursing homes"[MeSH Terms] OR ("nursing"[All Fields] AND "homes"[All Fields]) OR "nursing homes"[All Fields]) OR (("delivery of health care"[MeSH Terms] OR ("delivery"[All Fields] AND "health"[All Fields] AND "care"[All Fields]) OR "delivery of health care"[All Fields] OR "healthcare"[All Fields] OR "healthcare s"[All Fields] OR "healthcares"[All Fields]) AND ("setting"[All Fields] OR "setting s"[All Fields] OR "settings"[All Fields]))) AND ("ethiopia"[MeSH Terms] OR "ethiopia"[All Fields] OR "ethiopia s"[All Fields])) AND ((humans[Filter]) AND (2000/1/1:2023/12/31[pdat]) AND (english[Filter])). | 937 |
| **Scopus** | **Filters: Language: English; Date range: 01 January 2000 to 31 December 2023**  Prevalence* OR epidemiology* AND tuberculosis* OR *Mycobacterium tuberculosis** AND congregate* OR high-risk* OR prison* OR Prisoner* OR correctional* OR University* OR holy water settings OR Military* OR Refugee* OR Homeless* OR Nursing* OR Healthcare settings AND Ethiopia* AND PUBYEAR > 1999 AND PUBYEAR < 2024 AND ( LIMIT-TO ( LANGUAGE,"English" ) ) | 1015 |
| **ScienceDirect** | **Filters: Language**: English; **Date range:** 2000-2023  (Prevalence) AND (Tuberculosis) AND (Prisons OR refugee OR Universities OR Homeless shelters OR High-risk settings OR Spiritual holy water settings) AND Ethiopia | 2317 |
| **Google Scholar** | **Limits**: Item type: **Article**; Articles with all words; **Date range**: 01 January 2000 to 31 December 2023. 02 January 2024: 3: 57: 50PM  “Prevalence” AND “Pulmonary tuberculosis” OR “Tuberculosis” OR *Mycobacterium tuberculosis* OR *M.tuberculosis* AND “Congregate settings” OR “High-risk settings” OR “Prisons” OR Prisoners OR prison inmates OR correctional facilities OR “University” OR “Spiritual holy water sites” OR Military camps OR “Refugee camps” OR Homeless shelters OR Home for the elderly OR Nursing homes OR “Healthcare settings” AND “Ethiopia” | 4180 |

**S2 Table:** Quality appraisal results of included articles [1].

| **Author’s Name** | **Quality evaluation criteria** | | | | | | | | | **Quality Score** |
| --- | --- | --- | --- | --- | --- | --- | --- | --- | --- | --- |
|  | **a)** Was the sample frame appropriate to address the target population? | **b)** Were study participants sampled appropriately? | **c)** Was the sample size adequate? | **d)** Were the study subjects and the setting described in detail? | **e)** Was the data analysis conducted with sufficient coverage of the identified sample? | **f)** Were valid methods used for the identification of the condition? | **g)** Was the condition measured in a standard, reliable way for all participants? | **h)** Was there appropriate statistical analysis? | **i)** Was the response rate adequate, and if not, was the low response rate managed appropriately? |  |
| Moges *et al* (2012) [2] | Yes | Yes | Yes | Yes | Yes | Yes | Yes | Yes | NA | 8 |
| Abebe *et al* (2011) [3] | Yes | Yes | Yes | Yes | Yes | Yes | Yes | Yes | NA | 8 |
| Addis *et al* (2015) [4] | Yes | Yes | Yes | Yes | Yes | Unclear | Yes | Yes | Unclear | 7 |
| Bayu *et al* (2016) [5] | Yes | Yes | Yes | Yes | Yes | Unclear | Unclear | Yes | NA | 6 |
| Fuge *et al* (2016) [6] | Yes | Yes | No | Yes | Yes | Unclear | Unclear | Yes | NA | 5 |
| Zerihun *et al* (2014) [7] | Yes | Yes | No | Yes | Yes | Unclear | Yes | Yes | NA | 6 |
| Biadglegne *et al* (2014) [8] | Yes | Yes | Yes | Yes | Yes | Yes | Yes | Yes | Unclear | 8 |
| Ali *et al* (2015) [9] | Yes | Yes | Yes | Yes | Yes | Yes | Yes | Yes | Yes | 9 |
| Gebrecherkos *et al* (2016) [10] | Yes | Yes | Yes | Yes | Yes | Yes | Yes | Yes | Yes | 9 |
| Adane *et al* (2016) [11] | Yes | Yes | Yes | Yes | Yes | Yes | Yes | Yes | Unclear | 8 |
| Winsa *et al* (2015) [12] | Unclear | Yes | No | Yes | Yes | Yes | Unclear | Yes | NA | 5 |
| Gizachew *et al* (2017) [13] | Yes | Yes | Yes | Yes | Yes | Yes | Unclear | Yes | NA | 7 |
| Merid *et al* (2018) [14] | Yes | Yes | Yes | Yes | Yes | Yes | Yes | Yes | NA | 8 |
| Berihun *et al* (2018) [15] | Yes | Yes | No | Yes | Yes | Yes | Unclear | Yes | NA | 6 |
| Abayineh (2018) [16] | Yes | Yes | Yes | Yes | Yes | Yes | Unclear | Yes | NA | 7 |
| Agajie *et al* (2018) [17] | Yes | Yes | Yes | Yes | Yes | Yes | Unclear | Yes | NA | 7 |
| Tsegaye *et al* (2019) [18] | Yes | Yes | Yes | Yes | Yes | Yes | Yes | Yes | NA | 8 |
| Dibissa *et al* (2019) [19] | Yes | Yes | Yes | Yes | Yes | Unclear | Yes | Yes | NA | 7 |
| Adane *et al* (2019) [20] | Yes | Yes | Yes | Yes | Yes | Yes | Yes | Yes | NA | 8 |
| Duressa *et al* (2022) [21] | Yes | Yes | Yes | Yes | Yes | Unclear | Yes | Yes | NA | 7 |
| Dememew *et al* (2020) [22] | Yes | Yes | Yes | Yes | Yes | Yes | Yes | Yes | NA | 8 |
| Derseh *et al* (2017) [23] | Yes | Yes | Yes | Yes | Yes | Yes | Yes | Yes | Yes | 9 |
| Hordofa *et al* (2023) [24] | Yes | Yes | Yes | Yes | Yes | Yes | Yes | Yes | Yes | 9 |
| Meaza *et al* (2023) [25] | Yes | Yes | Yes | Yes | Yes | Yes | Yes | Yes | Yes | 9 |
| Mekonen *et al* (2018) [26] | Yes | Yes | Yes | Yes | Yes | Yes | Yes | Yes | NA | 8 |
| Mekonen *et al* (2016) [27] | Yes | Yes | Yes | Yes | Yes | Unclear | Yes | Yes | Unclear | 7 |
| Moges *et al* (2015) [28] | Yes | Yes | Yes | Yes | Yes | Unclear | Yes | Yes | NA | 7 |
| Semunigus *et al* (2016) [29] | Yes | Yes | Yes | Yes | Yes | Yes | Yes | Yes | NA | 8 |
| Shamebo *et al* (2023) [30] | Yes | Yes | Yes | Yes | Yes | Yes | Yes | Yes | Unclear | 8 |
| Shiferaw *et al* (2021) [31] | Yes | Yes | Yes | Yes | Yes | Yes | Yes | Yes | Yes | 9 |
| Wolde *et al* (2017) [32] | Yes | Yes | No | Yes | No | Yes | Yes | Yes | No | 6 |
| Legesse *et al (*2021) [33] | Yes | Yes | Yes | Yes | Yes | Yes | Yes | Yes | No | 8 |
| Reta *et al* (2023) [34] | Yes | Yes | Yes | Yes | Yes | Yes | Yes | Yes | No | 8 |
| Eyob *et al* (2002) [35] | Yes | Yes | No | Yes | No | Yes | Unclear | Yes | No | 5 |

**S3 Table:** The Egger’s publication bias test for small-study effects

| **Egger’s test**  Meta bias, egger  Effect-size label: Prevalence of PTB  Effect size: PREVALENCE  Std. err.: SE_PREVALENCE  Regression-based Egger test for small-study effects  Random-effects model  Method: REML  H0: beta1 = 0; no small-study effects  beta1 = 8.05  SE of beta1 = 1.434  z = 5.61  Prob > \|z\| = 0.0000 |
| --- |

**S4 Table:** The nonparametric trim-and-fill analysis of publication bias

| **Meta trim-and-fill analysis**  Effect-size label: Prevalence of PTB  Effect size: PREVALENCE  Std. err.: SE_PREVALENCE  Nonparametric trim-and-fill analysis of publication bias  Linear estimator, imputing on the left  Iteration Number of studies = 34  Model: Random-effects observed = 34  Method: REML imputed = 0  Pooling  Model: Random-effects  Method: REML  theta: Overall Prevalence of PTB   \| Studies \| theta \| [95% conf. interval] \| \| --- \| --- \| --- \| \| Observed \| 11.699 \| 7.972 - 15.427 \| \| Observed + Imputed \| 11.699 \| 7.972 - 15.427 \| |
| --- | --- | --- | --- | --- | --- | --- | --- | --- | --- |

**S1 Figure:** Funnel plot outcome for assessing publication bias. The prevalence of pulmonary TB is represented on the x-axis and the standard error (SE) of the prevalence of pulmonary TB is on the y-axis.

**S2 Figure**: The Galbraith plots of outcome, showing the assessment of heterogenicity across studies.

**References**

1. Munn Z, Moola S, Lis K, Riitano D, C T. Systematic reviews of prevalence and incidence. In: Aromataris E, Z. M, editors. *JBI Manual for Evidence Synthesis*: JBI; 2020.

2. Moges B, Amare B, Asfaw F, Tesfaye W, Tiruneh M, Belyhun Y, et al. Prevalence of smear positive pulmonary tuberculosis among prisoners in North Gondar Zone Prison, northwest Ethiopia. *BMC Infect Dis*. 2012;**12**:352.

3. Abebe DS, Bjune G, Ameni G, Biffa D, Abebe F. Prevalence of pulmonary tuberculosis and associated risk factors in Eastern Ethiopian prisons. *Int J Tuberc Lung Dis*. 2011;**15**(5):668-73.

4. Addis Z, Adem E, Alemu A, Birhan W, Mathewos B, Tachebele B, et al. Prevalence of smear positive pulmonary tuberculosis in Gondar prisoners, North West Ethiopia. *Asian Pac J Trop Med*. 2015;**8**(2):127-31.

5. Bayu B, Mekiso AB, Legesse T. Prevalence of pulmonary tuberculosis and associated factors among prisoners in Wolaita Zone, Southern Ethiopia: crosssectional study. *Am J Public Health Res*. 2016;**4**(4):142-8.

6. Fuge TG, Ayanto SY. Prevalence of smear positive pulmonary tuberculosis and associated risk factors among prisoners in Hadiya Zone prison, Southern Ethiopia. *BMC Res Notes*. 2016;**9**:201.

7. Zerdo Z, Medhin G, Worku A, Ameni G. Prevalence of pulmonary tuberculosis and associated risk factors in prisons of Gamo Goffa Zone, south Ethiopia: A cross-sectional study. *American Journal of Health Research*. 2014;**2**(5):291-7.

8. Biadglegne F, Rodloff AC, Sack U. A first insight into high prevalence of undiagnosed smear-negative pulmonary tuberculosis in Northern Ethiopian prisons: implications for greater investment and quality control. *PLoS One*. 2014;**9**(9):e106869.

9. Ali S, Haileamlak A, Wieser A, Pritsch M, Heinrich N, Loscher T, et al. Prevalence of Pulmonary Tuberculosis among Prison Inmates in Ethiopia, a Cross-Sectional Study. *PLoS One*. 2015;**10**(12):e0144040.

10. Gebrecherkos T, Gelaw B, Tessema B. Smear positive pulmonary tuberculosis and HIV co-infection in prison settings of North Gondar Zone, Northwest Ethiopia. *BMC Public Health*. 2016;**16**(1):1091.

11. Adane K, Spigt M, Ferede S, Asmelash T, Abebe M, Dinant GJ. Half of Pulmonary Tuberculosis Cases Were Left Undiagnosed in Prisons of the Tigray Region of Ethiopia: Implications for Tuberculosis Control. *PLoS One*. 2016;**11**(2):e0149453.

12. Winsa BB, Mohammed AE. Investigation on pulmonary tuberculosis among Bedele Woreda prisoners, Southwest Ethiopia. *International Journal of Biomedical Science and Engineering*. 2015;**3**(6):69-73.

13. Gizachew Beza M, Hunegnaw E, Tiruneh M. Prevalence and Associated Factors of Tuberculosis in Prisons Settings of East Gojjam Zone, Northwest Ethiopia. *Int J Bacteriol*. 2017;**2017**:3826980.

14. Merid Y, Woldeamanuel Y, Abebe M, Datiko DG, Hailu T, Habtamu G, et al. High utility of active tuberculosis case finding in an Ethiopian prison. *Int J Tuberc Lung Dis*. 2018;**22**(5):524-9.

15. Berihun YA, Nguse TM, Gebretekle GB. Prevalence of Tuberculosis and Treatment Outcomes of Patients with Tuberculosis among Inmates in Debrebirhan Prison, North Shoa Ethiopia. *Ethiop J Health Sci*. 2018;**28**(3):347-54.

16. Abayineh H. Prevalence and Determent Risk Factors of Active Pulmonary Tuberculosis in Federal Prison Administration High Security and Kilinto Appointment Prison Centers at Addis Ababa, Ethiopia. Addis Ababa University. 2018.

17. Agajie M, Disassa H, Birhanu M, Amentie M. Prevalence of pulmonary tuberculosis and associated factors in prisons of BenishangulGumuz region, Western Ethiopia. *Prevalence*. 2018;**6**(9).

18. Tsegaye Sahle E, Blumenthal J, Jain S, Sun S, Young J, Manyazewal T, et al. Bacteriologically-confirmed pulmonary tuberculosis in an Ethiopian prison: Prevalence from screening of entrant and resident prisoners. *PLoS One*. 2019;**14**(12):e0226160.

19. Dibissa KE, Waktole ZD, Tolessa BE. Prevalence of pulmonary tuberculosis and associated factors among prisoners in Western Oromia, Ethiopia: A cross-sectional study. *bioRxiv*. 2019:869727.

20. Adane K, Spigt M, Winkens B, Dinant GJ. Tuberculosis case detection by trained inmate peer educators in a resource-limited prison setting in Ethiopia: a cluster-randomised trial. *The Lancet Global health*. 2019;**7**(4):e482-e91.

21. Duressa TA, Mersha MA, Assefa D, Klinkenberg E. Prevalence and Associated Risk Factors of Pulmonary Tuberculosis Among Prisoners in Benishangul Gumuz Region, Ethiopia. 2022.

22. Dememew ZG, Jerene D, Datiko DG, Hiruy N, Tadesse A, Moile T, et al. The yield of community-based tuberculosis and HIV among key populations in hotspot settings of Ethiopia: A cross-sectional implementation study. *PLoS One*. 2020;**15**(5):e0233730.

23. Derseh D, Moges F, Tessema B. Smear positive pulmonary tuberculosis and associated risk factors among tuberculosis suspects attending spiritual holy water sites in Northwest Ethiopia. *BMC Infect Dis*. 2017;**17**(1):100.

24. Hordofa G, Mulatu G, Daka D. Prevalence, drug-susceptibility pattern and associated factors of Mycobacterium tuberculosis infection among prisoners in western Arsi zonal prisons, Oromia, South West Ethiopia. *IJID Regions*. 2023;**9**:1-6.

25. Meaza A, Yenew B, Amare M, Alemu A, Hailu M, Gamtesa DF, et al. Prevalence of tuberculosis and associated factors among presumptive TB refugees residing in refugee camps in Ethiopia. *BMC Infect Dis*. 2023;**23**(1):498.

26. Mekonnen A, Collins JM, Aseffa A, Ameni G, Petros B. Prevalence of pulmonary tuberculosis among students in three eastern Ethiopian universities. *Int J Tuberc Lung Dis*. 2018;**22**(10):1210-5.

27. Mekonnen A, Petros B. BURDEN OF TUBERCULOSIS AMONG STUDENTS IN TWO ETHIOPIAN UNIVERSITIES. *Ethiop Med J*. 2016;**54**(4):189-96.

28. Moges B, Amare B, Yismaw G, Workineh M, Alemu S, Mekonnen D, et al. Prevalence of tuberculosis and treatment outcome among university students in Northwest Ethiopia: a retrospective study. *BMC Public Health*. 2015;**15**:15.

29. Semunigus T, Tessema B, Eshetie S, Moges F. Smear positive pulmonary tuberculosis and associated factors among homeless individuals in Dessie and Debre Birhan towns, Northeast Ethiopia. *Ann Clin Microbiol Antimicrob*. 2016;**15**(1):50.

30. Shamebo T, Mekesha S, Getahun M, Gumi B, Petros B, Ameni G. Prevalence of pulmonary tuberculosis in homeless individuals in the Addis Ababa City, Ethiopia. *Frontiers in public health*. 2023;**11**:1128525.

31. Shiferaw MB, Sinishaw MA, Amare D, Alem G, Asefa D, Klinkenberg E. Prevalence of active tuberculosis disease among healthcare workers and support staff in healthcare settings of the Amhara region, Ethiopia. *PLoS One*. 2021;**16**(6):e0253177.

32. Wolde D, Tadesse M, Abdella K, Abebe G, Ali S. Tuberculosis among Jimma University Undergraduate Students: First Insight about the Burden of Tuberculosis in Ethiopia Universities-Cross-Sectional Study. *Int J Bacteriol*. 2017;**2017**:9840670.

33. Legesse T, Admenur G, Gebregzabher S, Woldegebriel E, Fantahun B, Tsegay Y, et al. Tuberculosis (TB) in the refugee camps in Ethiopia: trends of case notification, profile, and treatment outcomes, 2014 to 2017. *BMC Infect Dis*. 2021;**21**(1):139.

34. Reta Melese, Maningi Nontuthuko Excellent, Bernard Fourie P. Pulmonary tuberculosis (PTB) symptomatic individuals, their sociodemographic characteristics, genetic diversity and drug resistance patterns of Mycobacterium tuberculosis strains. University of Pretoria. [Dissertation]. Pretoria, South Africa: University of Pretoria. dataset: <https://doi.org/10.25403/UPresearchdata.22219072.v1>; 2023.

35. Eyob G, Gebeyhu M, Goshu S, Girma M, Lemma E, Fontanet A. Increase in tuberculosis incidence among the staff working at the Tuberculosis Demonstration and Training Centre in Addis Ababa, Ethiopia: a retrospective cohort study (1989-1998). *Int J Tuberc Lung Dis*. 2002;**6**(1):85-8.
