# Supplementary material for: Prevalence of pulmonary tuberculosis among key and vulnerable populations in hotspot settings of Ethiopia. A systematic review and meta-analysis
Source: PLoS One. 2024 Aug 29;19(8):e0309445. doi: 10.1371/journal.pone.0309445 (PMC11361443; doi:10.1371/journal.pone.0309445)
Supplement: Attachment — Submitted filename: Academic editor comment.docx [file pone.0309445.s003.docx]

Academic editor comment

***Prevalence of pulmonary tuberculosis among key populations in hotspot settings in Ethiopia: A systematic review and Meta-Analysis***

**Dear Authors,**

Congratulations for coming up with this quite relevant evidence of PTB among KVP. This is very important for Ethiopian TB program where the focus is on tailored active TB case finding is among KVPs at hotspot settings for TB.

**Major comment**

However, the is one critical comment to deal with. It is very instrumental to revise that all the literatures/references underwent for review were PTB. That is, it is advisable to check your topic/objective of assessing the prevalence PTB vs the cited references—looks some publication are not categorized in to form/types of TB,i.e not limited to PTB by excluding EPTB. If you are interested in only PTB that may be reflected clearly in the literature search and inclusion/exclusion criteria.

Minore commons

- The document will benefit out of professional English language editorial (grammar, structure and spacing) E.g university student vs University student; spacing in “..8.8% (95%CI: 5.00-12.55), University students 23.1% (95%CI: 15.81-30.37), Refugee camps 28.4%(95%CI: -1.27-58.15), Homeless individuals 5.8% (95%CI: -0.67- 12.35),..”
- Check the recently revised terminology ( under STOP TB KVP) as ‘key and vulnerable population’(KVPs) is the updated and standard terminology in use.
- You may use and refer the latest TB incidence estimate of TB in Ethiopia, 126/100k--[Global tuberculosis report 2023 (who.int)](https://www.who.int/publications/i/item/9789240083851) in the statement “ Ethiopia’s TB incidence was 157 per 100,000 population, in 2020 [5].” Consider changing the referenced in text and list as well.
- Check the explanation and justification of the declining in the prevalence of KVP. This might show the increasing effort of active TB screening after 2015 where the denominator, # screened KVPs, might be increasing.
- Review the manuscript write up per the PLOS ONE writing standard, e.g. TB cannot be defined twice- at introduction and discussion.
