# Supplementary material for: Prevalence of pulmonary tuberculosis among key and vulnerable populations in hotspot settings of Ethiopia. A systematic review and meta-analysis
Source: PLoS One. 2024 Aug 29;19(8):e0309445. doi: 10.1371/journal.pone.0309445 (PMC11361443; doi:10.1371/journal.pone.0309445)
Supplement: Attachment — Submitted filename: 2 Response to Reviewers.docx [file pone.0309445.s004.DOCX]

**Author's Response to Reviewers’ comments**

**Title**: Prevalence of pulmonary tuberculosis among key populations in hotspot settings of Ethiopia. A Systematic Review and Meta-Analysis

**Manuscript ID:** PONE-D-24-19020

PLOS ONE

**Dear reviewers**

First and foremost, we would like to extend our deepest gratitude to the reviewer(s)/editors for their time, effort, and commitment to reviewing our submitted paper and for their constructive comments. We greatly appreciate the detailed and thoughtful comments provided to our manuscript. These insightful comments were instrumental in guiding us to refine and improve our manuscript. They provided valuable perspectives that helped us address key issues and strengthen the overall presentation of our research. The detailed feedback highlighted areas that needed further clarification and allowed us to make necessary revisions, ultimately resulting in a more robust and impactful manuscript. We have made a rigorous effort to amend our manuscript in accordance with the reviewers'/editor’s comments and have employed additional revisions to further enhance the quality of our work. As highlighted in the main document, we made significant revisions in the manuscript. Hence, we made sure that each one of the comments has been addressed carefully and the manuscript is revised accordingly. Below are detailed responses (point-by-point responses) to all the comments/suggestions. All revisions that have been made to our manuscript are highlighted (trac changing) in the revised versions of the manuscript. Please let us know if you have further inquiries regarding the revised manuscript or if any queries or comments still need clarity. We will be happy to address them, now on time.

Thank you very much for your time and efforts to evaluate our manuscript.

With sincere regards,

Melese Abate Reta (PI)

Date: 03 August 2024

**Reviewer’s Comments and Author/s’ Responses**

**Major comment**

1. **Reviewer’s/editor’s Comment_1:**

However, there is one critical comment to deal with. It is very instrumental to revise that all the literatures/references underwent for review were PTB. That is, it is advisable to check your topic/objective of assessing the prevalence PTB vs the cited references—looks some publication are not categorized in to form/types of TB, i.e not limited to PTB by excluding EPTB. If you are interested in only PTB that may be reflected clearly in the literature search and inclusion/exclusion criteria.

**Author’s response_1:**

We greatly appreciate your valuable comments on this point. As we have clearly highlighted in the “title” of our paper, “inclusion criteria”, “search strategies”, and the “study outcomes” section, our main focus/objective is to assess the combined/pooled prevalence of PTB while excluding extrapulmonary TB (EPTB). We carefully constructed our search terms for PTB to ensure we captured all relevant papers from the electronic databases. However, if the eligible and included studies report both the incidence or prevalence of PTB and EPTB among presumptive TB patients, we carefully extract the data pertaining to PTB cases only and calculate it’s prevalence by excluding EPTB cases. We emphasize this statement in the “Study Outcomes” section of the manuscript. Additionally, in our **exclusion criteria**: (c) "Studies reporting only extrapulmonary TB cases or latent TB" were excluded.

**Minor Comments**

2. **Reviewer’s Comment_2:**

The document will benefit out of professional English language editorial (grammar, structure and spacing) E.g university student vs University student; spacing in “..8.8% (95%CI: 5.00-12.55), University students 23.1% (95%CI: 15.81-30.37), Refugee camps 28.4%(95%CI: -1.27-58.15), Homeless individuals 5.8% (95%CI: -0.67- 12.35),..”

**Author’s response_2:**

Thank you very much for your constructive comments. We have revised the paper to improve its writing, including grammar, structure, and spacing, throughout the manuscript. Additionally, we invited native English speakers to review our paper, resulting in many corrections.

3. **Reviewer’s Comment_3:**

Check the recently revised terminology (under STOP TB KVP) as ‘key and vulnerable population’(KVPs) is the updated and standard terminology in use.

**Author’s response_3:**

Thank you very much. We have reviewed the recently revised terminology for KVP and updated it to "key and vulnerable populations" (KVPs). These corrections have been made throughout the document.

4. **Reviewer’s Comment_4:**

You may use and refer the latest TB incidence estimate of TB in Ethiopia, 126/100k--[Global tuberculosis report 2023 (who.int)](https://www.who.int/publications/i/item/9789240083851) in the statement “ Ethiopia’s TB incidence was 157 per 100,000 population, in 2020 [5].” Consider changing the referenced in text and list as well.

**Author’s response_4:**

Thank you for your valuable comment. We have referred to the latest TB incidence estimates for Ethiopia from the Global Tuberculosis Report 2023 (126/100,000) and updated/changed the reference accordingly.

5. **Reviewer’s Comment_5:**

Check the explanation and justification of the declining in the prevalence of KVP. This might show the increasing effort of active TB screening after 2015 where the denominator, # screened KVPs, might be increasing.

**Author’s response_5:**

We have reviewed the explanation and justification for the declining trends in the prevalence of KVP and included the following statement in the discussion section: “Another possible reason for the decline in PTB prevalence post-2015 could be the country’s increased efforts in active TB screening after 2015. This might reflect a rise in the total number of screened populations, especially among key and vulnerable groups.”

6. **Reviewer’s Comment_6:**

Review the manuscript write up per the PLOS ONE writing standard, e.g. TB cannot be defined twice- at introduction and discussion.

**Author’s response_6:**

Thank you for this important comment. We have tried to adhere to the PLOS ONE manuscript writing standards and revised the introduction and discussion sections to remove the repeated use of the abbreviation "TB".
